# Comparative Physiological and Transcriptomic Analyses Reveal Mechanisms of Exogenous Spermidine-Induced Tolerance to Low-Iron Stress in *Solanum lycopersicum* L

**DOI:** 10.3390/antiox11071260

**Published:** 2022-06-27

**Authors:** Yu Shi, Yihong Zhao, Qi Yao, Feng Liu, Xiumin Li, Xiu Jin, Yi Zhang, Golam Jalal Ahammed

**Affiliations:** 1 College of Horticulture, Shanxi Agricultural University, Jinzhong 030801, China; shiyu@sxau.edu.cn (Y.S.); s20212238@stu.sxau.edu.cn (Y.Z.); z20203455@stu.sxau.edu.cn (Q.Y.); s20192167@stu.sxau.edu.cn (X.J.); 2 Research Institute of Vegetables, Hunan Academy of Agricultural Sciences, Changsha 410125, China; liufengrich@126.com (F.L.); M929989171@163.com (X.L.); 3 College of Horticulture and Plant Protection, Henan University of Science and Technology, Luoyang 471023, China

**Keywords:** polyamine, tomato, iron-deficiency, oxidative stress, transcriptomics

## Abstract

Iron (Fe) deficiency in plants is a major problem in agriculture. Therefore, we investigated both the physiological features and molecular mechanisms of plants’ response to low-Fe (LF) stress along with the mitigation of LF with exogenous spermidine (Spd) in tomato plants. The results showed that exogenous Spd foliar application relieved the suppressing effect of LF stress on tomato plants by regulating the photosynthetic efficiency, chlorophyll metabolism, antioxidant levels, organic acid secretion, polyamine metabolism and osmoregulatory systems. Analysis of transcriptomic sequencing results revealed that the differentially expressed genes of iron-deficiency stress were mainly enriched in the pathways of phytohormone signaling, starch and sucrose metabolism and phenyl propane biosynthesis in both leaves and roots. Moreover, Spd-induced promotion of growth under LF stress was associated with upregulation in the expression of some transcription factors that are related to growth hormone response in leaves (*GH3*, *SAUR*, *ARF*) and ethylene-related signaling factors in roots (*ERF1*, *ERF2*). We propose that traits associated with changes in low-iron-tolerance genes can potentially be used to improve tomato production. The study provides a theoretical basis for dealing with the iron deficiency issue to develop efficient nutrient management strategies in protected tomato cultivation.

## 1. Introduction

Iron (Fe) is a trace mineral element necessary for the normal life activities of almost all living organisms including plants. It is the fourth most abundant element in the earth’s crust. Despite the high total iron content in soils, the soluble iron (Fe^2+^) fraction is easily fixed to the insoluble form (Fe^3+^) in an alkaline environment, which seriously affects the normal uptake of iron by plants. Iron deficiency impairs photosynthetic efficiency, plant growth and biomass yield [1,2]. As a redox-active metal, Fe is engaged in photosynthesis, mitochondrial respiration, nitrogen anabolism, hormone (ethylene, gibberellic acid, jasmonic acid) synthesis and pathogen defense [3]. Iron also acts as the cofactor of many antioxidant enzymes, and thus iron deficiency has a regulatory effect on the antioxidant mechanisms, including the activities of superoxide dismutase (SOD), peroxidase (POD) and catalase (CAT) in plants [4], which are in charge of protecting the biological system against the harmful effects of reactive oxygen species (ROS) [5]. ROS are produced in all forms of aerobic life under stress or normal conditions. The excessive production of ROS causes oxidative damage that has a negative impact on the function of important macromolecules [6]. Thus, a better understanding of the mechanisms of plant response to iron stress can be useful to improve crop stress resilience and enhance crop yield and quality [7].

When plants are exposed to a low-Fe environment, insufficient iron uptake causes retarded growth, interveinal chlorosis and reduced plant fitness. In severe iron deficiency, chloroplasts are dissociated or vesiculated, thus affecting chlorophyll formation [8]. To ensure the normal growth of plants, phytohormone auxin accumulates in large amounts in the roots, promoting the development of lateral roots and positively regulating the transcriptional expression of the *FIT1* and *AHA2* genes. Thus, growth factors are involved in the plant Fe-deficiency response network through different pathways [9].

Under low-iron stress, plants enhance Fe uptake by the root system through two strategies: One strategy based on the reduction that occurs in all dicotyledons and non-grass monocotyledons, called strategy I, and another strategy relying on chelation, which is limited to monocotyledons, called strategy II [10]. Strategy-II plants produce plant iron carriers capable of chelating Fe^3+^, which are then absorbed by specific epidermal root cell plasma membrane transporters [11]. In tomatoes, on the one hand, as Strategy-I species, acidification of the root mesenchyme by plasma membrane H^+^-ATPase activity occurs to dissolve Fe^3+^, and reduction to Fe^2+^ by Fe^3+^-chelating reductase (FCR) activity increases iron solubility. Afterward, translocation of the resulting Fe^2+^ to the root cell via a specific Fe transporter (*IRT1*) takes place to accomplish iron acquisition in plants [12]. On the other hand, nitric oxide (NO) accumulates in the roots and promotes the expression of *FER/FIT*, as well as *IRT* and *FRO* genes, thus participating in the response to iron-deficiency stress in plants [13].

Polyamines are highly bioactive, low-molecular-weight aliphatic amines that occur as ubiquitous secondary metabolites in plants. Polyamines can bind to phospholipids and other biomolecules with negatively charged groups of nucleic acids and proteins through their ionic and hydrogen bonds, which widely affect the biological activity of plants [14]. In previous research reports, polyamines have been shown to perform an extremely important role in alleviating plant stress. Among the three widely distributed major polyamines, spermidine (Spd) plays a crucial role in abiotic stress tolerance. Due to its multivalent cation property, its physiological function is stronger and more associated with stress tolerance in plants [15]. Spd is a common polyamine in plants and is involved in adaptations to salinity [16], drought [17], cold [18] and heavy metals [19]. Some studies have shown that Spd modulates antioxidant enzyme activity and the expression of related genes in tomato seedlings exposed to high temperatures [20]. Exogenous Spd has been found to play an important role in remediating the effects of environmental stress on plants [21]. However, to date, few studies have reported on the Spd-mediated tolerance to iron stress, particularly in tomato plants.

In the present research, using ‘Micro-tom’ tomato as the object of study, we explored the effect of exogenously sprayed Spd on the growth, physiology and metabolism of tomato seedlings under low-Fe stress. The physiological analysis, combined with transcriptomic analyses, shed new light on the mechanism of Spd-mediated low-Fe tolerance in tomato seedlings from both physiological and molecular perspectives, which provides a theoretical basis for improving the uptake and utilization of Fe in protected cultivation.

## 2. Materials and Methods

### 2.1. Plant Materials, Growth Conditions and Experimental Treatments

Tomato (*Solanum lycopersicum* L.) cv ‘Micro-Tom’ seeds were purchased from the Ball Horticulture Company (West Chicago, IL, USA). Healthy seeds were selected and sown on a petri dish with distilled water. The germinated seedlings were transferred to 72-well trays and cultured under artificial climate chamber conditions: temperature 28 °C/22 °C (14 h day/10 h night), humidity 80% and light intensity 600 μmol m^−2^s^−1^. When the plants had four fully expanded leaves, uniformly grown tomato seedlings were planted in a hydroponic tank filled with half-strength Japanese Yamazaki tomato formula nutrient solution [22]. After five days of seedling culture in hydroponics, the following treatments were applied: (1) CK (control), Yamazaki formula nutrient solution (Fe concentration was 100 μM); (2) LF, low-Fe nutrient solution (Fe concentration was 10 μM); (3) Spd, Yamazaki formula nutrient solution (100 μM Fe) + 0.25 mM Spd foliar spray; (4) LF + Spd, low-Fe (10 μM) nutrient solution + 0.25 mM Spd foliar spray. The Spd was purchased from the Beijing Solarbio Technology Company. Both sides of the tomato leaves were sprayed with freshly prepared Spd solution (approximately 10 mL per plant). Low-Fe stress was imposed 1d after the Spd treatment. Foliar-spraying of Spd was repeated every two days. The control tomato plants were foliar-sprayed with an equal volume of distilled water. The nutrient solution was changed every three days, the pH value was adjusted to 6.0 ± 0.2 and an intermittent supply of oxygen was provided using an aeration pump. On the 10th day of treatments, unless otherwise stated, samples were collected/used for various analyses such as photosynthetic fluorescence indicators, osmoregulatory substance content, organic acid and polyamine contents and RNA sequencing. Biomass measurements were performed on day 15 of treatment.

### 2.2. RNA-Seq Analysis and Quantitative Real-Time PCR Analysis

Transcriptome sequencing was performed on samples from four treatments—CK, Spd, LF and LF + Spd—collected on day 10 of treatments by Hangzhou Lianchuan Biological Technology Co., Ltd. RNA-seq was performed with three biological replicates for each treatment. All raw sequencing data from the current study were deposited into the NCBI database under the accession number “PRJNA834903” (https://www.ncbi.nlm.nih.gov/sra/PRJNA834903), (accessed on 4 May 2022). Analysis of significant differences between samples was performed using R packages edgeR or DESeq2. Genes with differential fold FC > twofold or FC < 0.5-fold and a *p*-value < 0.05 were defined as differentially expressed genes [23]. GO (Gene Ontology) enrichment and KEGG (Kyoto Encyclopedia of Genes and Genomes) pathway enrichment were analyzed using the clusterProfiler R package. GO functional enrichment and KEGG pathway analysis were performed by Goatools (https://github.com/tanghaibao/Goatools), (accessed on 6 June 2022) and KOBAS (http://kobas.cbi.pku.edu.cn/home.do), (accessed on 6 June 2022). The qRT-PCR test reaction system and primers used for qRT-PCR are shown in Supplementary Tables S1 and S2, respectively. Samples were added to a 96-well plate and then reacted in an Applied Biosystems Quant Studio three real-time fluorescence quantitative PCR system (QuantStudio 3, ThermoFisher Scientific™, Waltham, MA, USA). The qRT-PCR amplification procedure consisted of Stage 1: pre-denaturation, one cycle 95 °C, 30 s; Stage 2: PCR reaction, 40 cycles of 95 °C for 10 s, 60 °C for 30 s, 72 °C for 40 s. Relative gene expression was estimated using the 2^-∆∆Ct^ method [24]. qRT-PCR experiments were performed in biological triplicates.

### 2.3. Determination of Biomass and Root Morphology, Root Vigor and Root Fe^3+^ Reductase Activity

After 15 days of treatment, five seedlings were randomly picked from each treatment, and the selected plants were cut from the same part, divided into above-ground and below-ground parts, any water on the plant surface was dried with absorbent paper and the fresh weight was measured. The samples were then placed in an electric thermostatic drying oven (Heratherm™ General Protocol Ovens, 51028148, Thermo Scientific™, Waltham, MA, USA) set to 105 °C for 30 min. After adjusting the temperature to 80 °C, the material was dried to a constant weight before measuring the dry weight. For root morphology measurements, the whole root system of a plant was scanned with a root system scanner (Epson Perfection V800 Photo, B11B223201, Epson America, Inc., Los Alamitos, CA, USA). The analysis was done using a root scanner (WinRhizo PRO, version 2017, Regent Instruments Inc., Quebec City, QC, Canada), and parameters such as the total root length, total surface area, total volume and average diameter were read [25]. Root vigor was determined by the triphenyl tetrazolium chloride (TTC) method [26]. Fe^3+^ reductase activity was determined according to the method of Ekmekcioglu C [27]. Three biological replicates for each treatment were set in treatments.

### 2.4. Determination of Photosynthetic Pigment Content and Photosynthetic Index

On the 10th day, chlorophylls (Chl) such as Chla, Chlb and carotenoids were measured in the third fully-expanded leaf [28]. About 0.1 g of leaf tissue was placed in a tube containing 96% ethanol in the dark for about 24 h until the leaves turned completely white. The absorbance values of chlorophyll extracts at 470 nm, 649 nm and 665 nm were measured with a spectrophotometer (UV-2450, Shimadzu, Kyoto Prefecture, Japan), and chlorophyll a, chlorophyll b and carotenoid contents were calculated.

The photosynthetic indexes such as the net photosynthetic rate (Pn), transpiration rate (Tr), intercellular CO_2_ concentration (Ci) and stomatal conductance (Gs) were measured using a portable photosynthetic apparatus (LI-6800, Li-COR Inc., Lincoln, NE, USA) on a clear day at around 10 a.m. The parameters were set to a flow rate of 500 μmol·s^−1^, leaf temperature of 28 °C and CO_2_ concentration of 400 µmol·mol^−1^; a CO_2_ cylinder was used to stabilize the CO_2_ environment [22].

Following 24 h of darkness, the seedling leaves were sampled to test the maximum photochemical efficiency, i.e., Fv/Fm [29]. In addition, the actual photochemical efficiency of PSⅡ (ΦPSⅡ), photosynthetic electron transfer rate (ETR), photochemical quenching coefficient (qP) and non-photochemical quenching coefficient (NPQ) were measured after 30 min of plant exposure to natural light conditions [30].

### 2.5. Determination of Antioxidant Properties and Osmoregulatory Substances

A fresh-leaf or root sample (0.3 g) was placed in a pre-cooled pestle and mortar and ground to a fine frozen powder under liquid nitrogen, followed by homogenization in 3 mL 50 mM phosphate buffer (pH 7.8) in an ice bath. Then, homogenate centrifugation was done at 12,000× *g* for 15 min at 4 °C. The supernatant was used to determine the peroxidase (POD) [31], catalase (CAT) [32] and superoxide dismutase (SOD) [33] activity. Activity analyses of POD, CAT and SOD were performed as described previously [34]. Three biological replicates for each treatment were performed. The lipid peroxidation level was measured by estimating the malondialdehyde (MDA) content in roots using thiobarbituric acid (TBA) [35]. Electrolyte leakage (%) was estimated by measuring ion leakage from roots according to the method of Shou [36]. The roots (which weighed 0.1 g) were placed in centrifuge tubes, then each tube was filled with 20 mL of distilled water. The conductivity (A1) was first measured after shaking the tube well, then the conductivity (A2) was again measured after shaking the tube in the shaker for 2 h. Finally, the sample was boiled and cooled to room temperature to measure the conductivity (A3). Relative electrolyte leakage was measured as follows: Relative conductivity = (A2−A1)/(A3−A1). The content of H_2_O_2_ in leaves and roots was determined by the method of Willekens [37]. The content of O^2•−^ in leaves and roots was analyzed by the method previously described by Li et al. [38]. Proline and soluble protein contents were determined by the methods of Bates [39] and Bradford [40], respectively. Meanwhile, the free amino acids and soluble sugar contents were determined by the method of Zhang et al. [41]. Each treatment was repeated three times to ensure the reliability of the results. The organic acid content was determined by high-performance liquid chromatography [42]. Parameter settings were as follows: a ZORBAX Eclipse XDB-C18 column (4.6 × 250 mm, 5 mm) was used; the mobile phase was set at 0.04 mol·mL^−1^, pH 2.4, KH_2_PO_4_-H_3_PO_4_ buffer solution; the flow rate was 0.8 mL·min^−1^; the column temperature was 30 °C, the detection wavelength was 210 nm and the injection volume was 10 µL.

### 2.6. Determination of Sucrose Content and Metabolism-Related Enzyme Activities

The sucrose content was determined by the hydrochloric acid-resorcinol method previously described by Zhang et al. [43]. We accurately weighed 0.1 g of leaves and roots and took 0.2 and 0.4 mL of supernatants, respectively. After adding 200 µL NaOH, the solution was boiled for 5 min at 100 °C, then cooled, before 2.8 mL 30% HCL and 0.8 mL 0.1% resorcinol were added, with the contents shaken well. Then, they were placed in a water bath at 80 °C for 10 min for the reaction to occur, and after cooling, the OD value was measured at 480 nm. Three replicates of each treatment were performed. Standard curves with different concentration gradients of sucrose were prepared with the standard solution and used to calculate the actual sucrose content in leaves and roots. To analyze the activities of sugar metabolism-related enzymes, frozen samples of leaves were weighed to 0.1 g. Sucrose synthase (SS), sucrose phosphate synthase (SPS), acid convertase (AI) and neutral convertase (NI) activities were determined using the corresponding enzyme activity assay kits (Beijing Solarbio Science & Technology Co., Ltd., Beijing, China).

### 2.7. Determination of Polyamine Content

Polyamines extraction from tomato seedlings was performed using the methods described by Flores and Galston [44]. The content of polyamines was determined by HPLC (high-performance liquid chromatograph UltiMate3000, ThermoFisher Scientific™, Waltham, MA, USA). The instrumentation and settings for endogenous polyamine analyses were as follows: a ZORBAX Eclipse XDB-C18 column (4.6 × 250 mm, 5 mm) and mobile phase (methanol: acetonitrile: water = 58:2.5:39.5) were used with a detection wavelength of 230 nm, flow rate of 1 mL·min^−1^, column temperature of 30 °C and injection volume of 10 µL. The organic solvents used above were of chromatographic-grade purity and the water was ultrapure. The mobile phase was configured for use after ultrasonic sonication beforehand.

### 2.8. Statistical Analysis

All data were subjected to analysis of variance (ANOVA), analyzed with SPSS 21.0 statistical software and plotted with Microsoft Excel 2016. For multiple mean comparisons, differences between treatment means were separated by Duncan’s multiple range test at *p* < 0.05.

## 3. Results

### 3.1. Overview of Sequencing Data-Quality Control

In this experiment, the leaves and roots of the Control (CK), Low Fe (LF), Spermidine (Spd) and Low Fe + Spd (LFS) were sequenced, and each treatment was replicated three times. The results showed that 99.98% of the nucleotides in the transcriptome sequencing data reached Q20, and 97.25% of the nucleotides exceeded Q30 (Table 1).

### 3.2. Analysis of Differentially Expressed Genes

**Figure 1:**
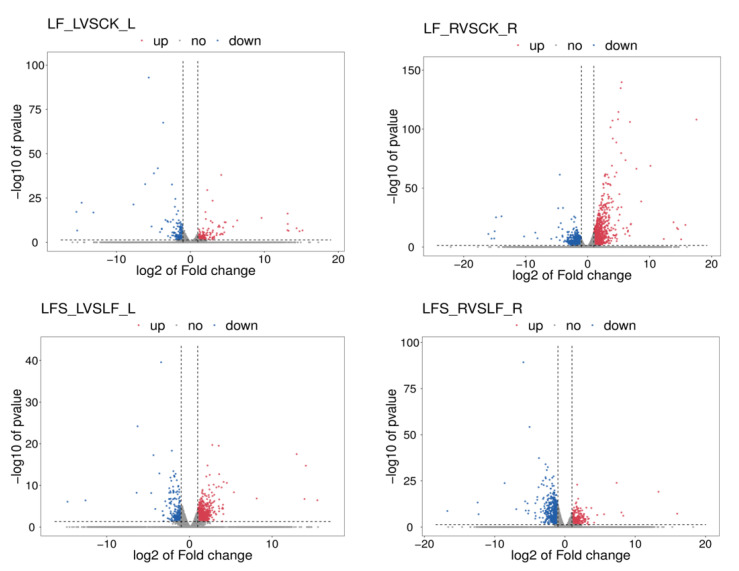
Volcano maps of expression differences. The horizontal coordinate represents the different expression fold changes of the gene in different samples, and the vertical coordinate represents the statistical significance of the difference in the gene expression change. LF_L vs. CK_L, Low Fe_Leaf sample vs. Control_Leaf sample; LF_R vs. CK_R, Low Fe_Root sample vs. Control_Root sample; LFS_L vs. LF_L, Low Fe + Spd_Leaf sample vs. Low Fe_Leaf sample; LFS_R vs. LF_R, Low Fe + Spd_Root sample vs. Low Fe_Root sample.

To get a closer look at the differentially expressed genes, we mapped volcanoes (Figure 1). In the volcano maps, red represents significantly upregulated differently expressed genes, blue represents significantly downregulated differently expressed genes and gray represents non-significant differently expressed genes.

**Figure 2:**
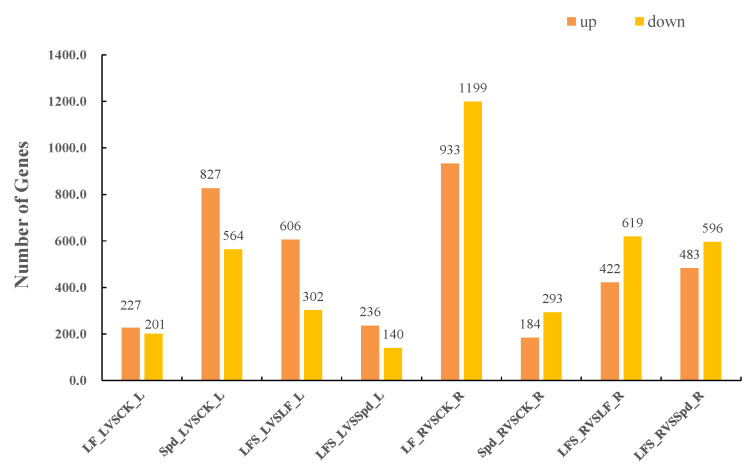
Number of significantly differentially expressed genes in different treatments. LF_L vs. CK_L, Low Fe_Leaf sample vs. Control_Leaf sample; Spd_L vs. CK_L, Spd_Leaf sample vs. Control_Leaf sample; LFS_L vs. LF_L, Low Fe + Spd_Leaf sample vs. Low Fe_Leaf sample; LFS_L vs. Spd_L, Low Fe + Spd_Leaf sample vs. Spd_Leaf sample; LF_R vs. CK_R, Low Fe_Root sample vs. Control_Root sample; Spd_R vs. CK_R, Spd_Root sample vs. Control_Root sample; LFS_R vs. LF_R, Low Fe + Spd_Root sample vs. Low Fe_Root sample; LFS_R vs. Spd_R, Low Fe + Spd vs. Spd_Root sample.

FPKM (fragments per kilobase of exon model per million mapped fragments) was used to count the expression abundance of known genes in different samples. In this experiment, we used the difference multiplier FC ≥ 2 or FC ≤ 0.5 (i.e., the absolute value of log2FC ≥ 1) as the threshold of change and a *p*-value <0.05 as the criterion for screening differential genes. The number of differentially expressed genes in each comparison group was counted, and a bar chart (Figure 2) was used to visualize the number of significantly differentially expressed genes in different comparison groups, as well as the specific changes (up- and downregulation). Compared to the control, 227 genes were upregulated and 201 genes were downregulated in the low-iron-treated leaves (LF), whereas the number of differentially expressed genes was higher in the root system, where 933 genes were upregulated and 1199 genes were downregulated, which indicated that the low-iron treatment had a more profound effect on transcription in the root system than in the leaves. Again, compared to the LF treatment, 606 genes were upregulated in the LF + Spd-treated leaves, and 302 genes were downregulated, while 422 genes were upregulated and 619 genes were downregulated in the root sample.

Then, we performed Venn diagram analysis for Control vs. Low Fe (CK vs. LF) and Low Fe vs. Low Fe + Spd (LF vs. LFS), gene ontology (GO) enrichment for Low Fe+ Spd_Leaf sample vs. Low Fe_Leaf sample (LFSL vs. LFL) and Low Fe + Spd_Root sample vs. Low Fe_Root sample (LFSR vs. LFR) and KEGG (Kyoto Encyclopedia of Genes and Genomes) pathway enrichment analysis for differently expressed genes as influenced by Spd treatment.

**Figure 3:**
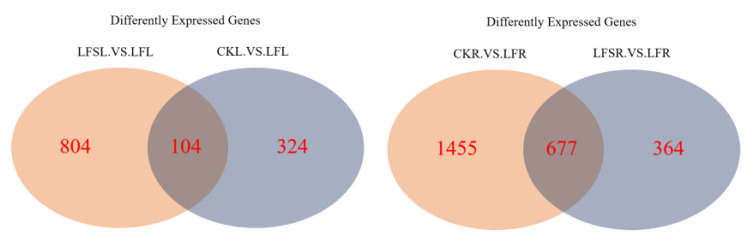
Venn diagram of significantly differentially expressed genes in different treatment comparisons. LFSL. vs. LFL, Low Fe+ Spd_Leaf sample vs. Low Fe_Leaf sample; CKL. vs. LFL, Control_Leaf sample vs. Low Fe_Leaf sample; CKR.VS.LFR, Control_Root sample vs. Low Fe_Root sample; LFSR. vs. LFR, Low Fe+ Spd_Root sample vs. Low Fe_Root sample.

The Venn diagram can visualize not only the number of differently expressed genes in the different treatment groups but also the number of genes that are differently expressed in each treatment group in total. As shown in Figure 3, a total of 104 differently expressed genes were co-expressed among 428 differentially expressed genes in the treatment group CK vs. LF, and there were 908 differentially expressed genes in the treatment group LFS vs. LF in the leaves. In the case of the root sample, a total of 677 differently expressed genes were co-expressed among 2132 differently expressed genes in the treatment group CK vs. LF, and there were 1041 differently expressed genes in the treatment group LFS vs. LF.

**Figure 4:**
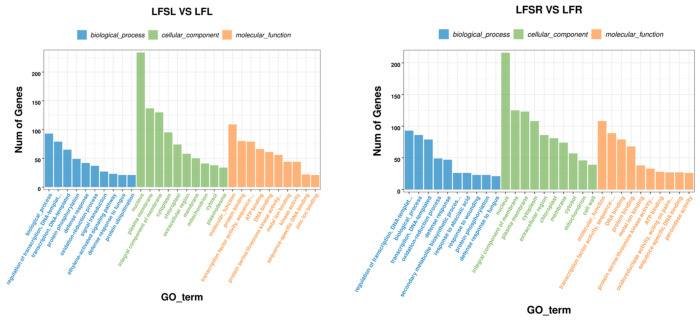
GO enrichment analysis of differently expressed genes. The abscissa represents different GO terms, blue represents biological processes, green represents cellular components, orange represents molecular functions and ordinate represents the number of differentially expressed genes. LFSL vs. LFL, Low Fe + Spd_Leaf sample vs. Low Fe_Leaf sample; LFSR vs. LFR, Low Fe + Spd_Root sample vs. Low Fe_Root sample.

The GO enrichment analysis of LFS vs. LF showed that enrichment in biological processes (BP) was mostly in functions such as transcriptional regulation with DNA as the template, protein phosphorylation, defense responses, redox processes, signal transduction processes, ethylene-activated signaling pathways, defense responses against fungi, and protein ubiquitination (Figure 4). In cellular components (CC), differentially expressed genes were involved in biological functions such as those of the nucleus, plasma membrane, membrane components, cytoplasm, chloroplast and extracellular regions. In molecular functions (MF), they were mainly enriched in sequence-specific protein-binding, specific DNA sequence-binding transcription factor activity, ATP-binding, DNA-binding, protein serine/threonine kinase activity, and metal ion-binding.

**Figure 5:**
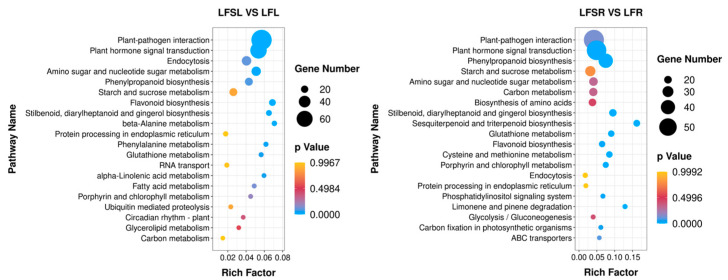
Enrichment analysis of differentially expressed genes in the KEGG pathway. The horizontal axis indicates the degree of enrichment (Rich factor), and the vertical axis indicates the enriched KEGG pathway; the size of the dots indicates the number of differentially expressed genes enriched in a KEGG pathway; the color of the dots indicates different *p* values; the Rich factor indicates the number of differentially expressed genes belonging to a KEGG pathway/the total number of genes belonging to this KEGG pathway. The larger the Rich factor, the higher the enrichment of the KEGG pathway. LFSL vs. LFL, Low Fe + Spd_Leaf sample vs. Low Fe_Leaf sample; LFSR vs. LFR, Low Fe + Spd_Root sample vs. Low Fe_Root sample.

To further explore the most important biochemical/metabolic pathways and signal transduction pathways involved in differentially expressed genes due to Spd treatment in low-iron-supplied tomato plants, the top-20 significantly enriched pathways were screened for KEGG enrichment analysis by the number of genes enriched in this pathway, and the enrichment results are presented in the form of bubble plots (Figure 5). KEGG enrichment analysis was performed on 908 differentially expressed genes in leaves and 1041 differentially expressed genes in roots, comparing the low-iron treatment and combined treatment of Spd and low iron.

The results showed that a total of 707 differently expressed genes in leaves were significantly enriched in 113 KEGG metabolic pathways, concentrated in metabolic pathways such as plant-pathogen interaction (79), phytohormone signaling (61), cytokinesis (30), amino and nucleotide sugar metabolism (29), phenyl propane biosynthesis (23) and starch and sucrose metabolism (23). A total of 867 differently expressed genes were significantly enriched in 117 KEGG metabolic pathways in the root system, concentrated in metabolic pathways such as plant-pathogen interaction (58), phytohormone signaling (56), benzyl propane biosynthesis (40), starch and sucrose metabolism (28), amino and nucleotide sugar metabolism (23) and carbon metabolism (22). It was found that the differentially expressed genes of iron-deficiency stress were mainly enriched in the pathways of phytohormone signaling, starch and sucrose metabolism and phenyl propane biosynthesis in both leaves and roots.

**Figure 6:**
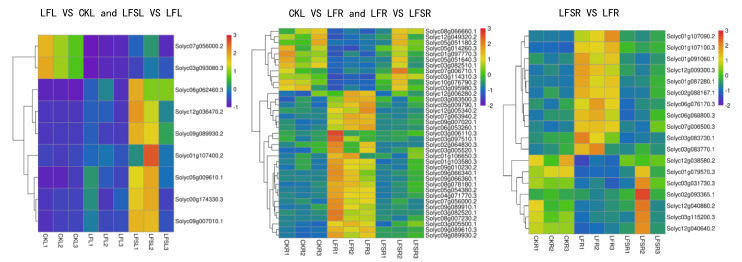
Heat map of differentially expressed genes related to plant hormone signal transduction and sucrose metabolism. Low Fe + Spd_Root sample vs. Low Fe_Root sample.

The results of GO enrichment analysis and KEGG pathway enrichment analysis showed that the differentially expressed genes in plant hormone signaling processes and starch and sucrose metabolism were significantly affected by low-Fe stress. Therefore, we performed a heat map analysis of differentially expressed genes in phytohormone signaling pathways and starch and sucrose metabolism. The results showed that a total of nine differently expressed genes in the phytohormone signal transduction pathway—*Solyc00g174330.3(PR1)*, *Solyc05g009610.1(GID1)*, *Solyc09g007010.1(PR1)*, *Solyc06g062460.3(PIF3)*, *Solyc07g056000.2(TCH4)*, *Solyc09g089930.2(ERF1)*, *Solyc12g036470.2(PIF3)*, *Solyc01g107400.2(GH3)* and *Solyc03g093080.3(TCH4)*—were common to leaves in the comparison groups of LF vs. CK and LFS vs. LF (Figure 6). The expression of oleuropein sterol regulatory protein EBRU1 precursors *Solyc07g056000.2(TCH4)* and *Solyc03g093080.3(TCH4)* was downregulated under low-iron treatment, while all other genes were upregulated. In contrast, all nine differently expressed genes were upregulated in leaves after spraying with Spd under low-iron treatment. A total of 37 differently expressed genes were expressed in the root system, mostly concentrated in the growth hormone and ethylene metabolic pathways. The expression of *Solyc03g082510.1(SAUR)* and *Solyc10g076790.2(AUX1)* in the growth hormone metabolic pathway and *Solyc08g066660.1(ERF1)* and *Solyc03g114310.3(CTR1)* in the ethylene metabolic pathway were downregulated under low-Fe stress, while their expression with Spd treatment under low-iron stress was upregulated. It appears that differentially expressed genes related to hormone metabolism showed different trends in leaves and roots. For the upregulated genes, in leaves, Spd foliar-spray treatment could further upregulate gene expression, whereas, in roots, Spd foliar treatment downregulated genes to the control level. Of the 18 differentially expressed genes in the root system for starch and sucrose metabolic processes, seven differently expressed genes were downregulated and 11 differently expressed genes were upregulated, and the expression of genes related to hormone signaling was consistent with the Spd treatment; nonetheless, all of these were backregulated to the control level in the root sample.

**Figure 7:**
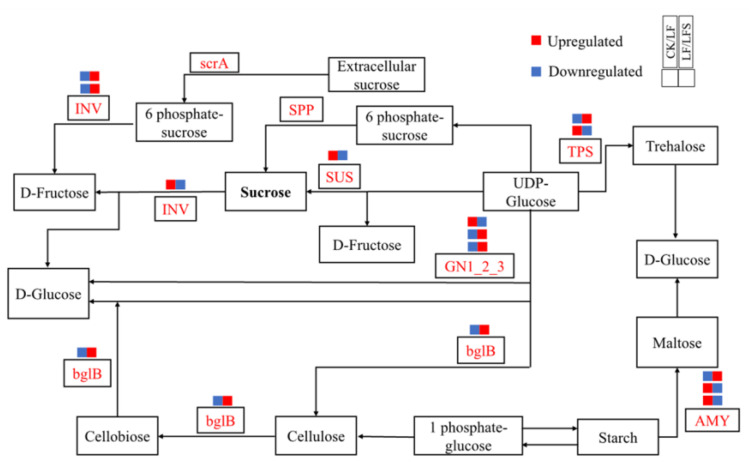
Diagram of plant sucrose metabolism pathway.

Then, we analyzed the expression of genes related to hormone signaling pathways and sucrose metabolism, as well as differentially expressed genes of other metabolic pathways, as shown in Figure 7. In leaf blades, *Solyc01g008620.3(GN1-2-3)* expression was upregulated in the starch and sucrose metabolic pathways, which potentially accelerated glucose synthesis; *Solyc02g071620.3(CHLP)* and *Solyc07g064720.3(CHLP)* expression were upregulated in porphyrin and chlorophyll metabolism, which in turn, potentially functioned in the synthesis of chlorophyll a and chlorophyll b, respectively. *Solyc07g024000.3(NOL)* expression was downregulated, thus, perhaps, inhibiting the conversion of chlorophyll b to hydroxy-chlorophyll a. In the photosynthetic pathway, *Solyc11g006910.2(PetF)* iron oxytocin gene expression was upregulated during photosynthetic electron transfer; in the peroxisome pathway, i.e., the antioxidant enzyme system, *Solyc12g094620.2(CAT)* expression was upregulated in the antioxidant enzyme system and so on. In the root system, more genes are related to the expression of hormone metabolism, and among them, the expression of *Solyc10g076790.2(AUX1)* and *Solyc03g082510.1(SAUR)* was upregulated after Spd treatment under low iron, both of which are jointly involved in plant cell growth. However, *Solyc09g089610.3(ETR)*, *Solyc09g066360.1(ERF1)* and *Solyc04g071770.3(ERF2)* transcripts were downregulated, which potentially alleviated the effect of ethylene on cell senescence. Again, *Solyc12g038580.2(TPS)* expression was upregulated in the starch and sucrose metabolic pathways, which affected sugar synthesis, and *Solyc12g009300.3(SUS)* expression was downregulated, which might affect sucrose synthase activity. In the peroxisome pathway, the epoxidation process was promoted by upregulation of *Solyc01g066457.1(EPHX2)*. Upregulation of *Solyc01g058210.2(HMGCL)*, *Solyc10g007600.3(HAO)* and *Solyc12g099930.2(AGXT)* contributed to amino acid metabolism, and upregulated expression of *Solyc12g094620.2(CAT)* in hydrogen peroxide metabolism potentially increased redox levels. In addition to affecting the expression of related metabolic genes in each pathway, Spd-spraying under low iron upregulated the expression of *Solyc02g069200.3(IRT1)*, *Solyc01g094890.3((FRO2)* and *Solyc01g094910.3(FRO),* which potentially improved the Fe uptake and transport capacity of the root system under low-iron stress.

Finally, expression trends of six selected differentially-expressed genes related to iron transport or sucrose metabolism in the root were validated by qRT-PCR. The trends for the gene expression in qRT-PCR (Supplementary Figure S1) were approximately the same as the transcriptome sequencing results, indicating that the results were credible.

### 3.3. Exogenous Spd Improved the Growth and Photosynthetic Efficiency of Tomato Plants under Low-Iron Stress

The growth of tomato seedlings was significantly inhibited by low-Fe stress, along with significantly decreased dry and fresh weights by 28.57 and 27.91%, respectively. However, plant biomass was significantly increased by Spd foliar treatment under low-iron stress (Table 2). Likewise, root growth was significantly affected by low-iron stress, but Spd foliar treatment under low-Fe conditions increased the total root length, total root surface area and total root volume by 78.63, 41.35 and 40.91%, respectively, compared to the low-iron treatment (Table 2). It is evident that exogenous Spd-spraying has a mitigating effect on the growth of tomato seedlings under low-iron stress.

Moreover, root vigor and Fe^3+^ reductase activity were significantly increased by either low-iron stress or Spd-spraying (Supplementary Figure S2). When compared to the low-Fe treatment, root vigor and Fe^3+^ reductase activity were further increased by 23.21 and 21.35%, respectively, after spraying with Spd under low-Fe stress.

The photosynthetic pigment content of tomato leaves was repressed by low-iron stress. However, the chlorophyll a, chlorophyll b and chlorophyll a + b contents were significantly increased by 23.58, 12.50 and 21.58%, respectively, in Spd treatment under low-Fe stress compared to low-iron stress only, though the carotenoid content was affected by Spd treatment under low-iron stress (Table 3).

In line with the photosynthetic pigment concentrations, the net photosynthetic rate was inhibited by 49.07% in leaves under low-Fe stress, and exogenous foliar-spraying of Spd alleviated the reduction of gas-exchange parameters in tomato leaves caused by low-Fe stress, and increased Pn, Tr, Gs and the intercellular CO_2_ concentration (Ci) (Table 4).

Under low-Fe stress, chlorophyll fluorescence parameters such as the maximum photochemical efficiency of PSII (Fv/Fm), electron transfer efficiency (ETR), actual photochemical quantum yield of PSII (ΦPSII) and photochemical quenching coefficient (qP) of leaves significantly decreased by 7.47, 37.21, 37.32 and 35.47%, respectively, and the non-photochemical quenching coefficient (NPQ) increased by 85.94%. However, all these indicators, except for qP, increased significantly after spraying with Spd, suggesting that exogenous foliar-spraying with Spd under low-iron stress had a strong ameliorative effect on leaf chlorophyll fluorescence characteristics (Table 5).

### 3.4. Effect of Exogenous Spd on ROS Accumulation, Antioxidant System and Osmoregulatory Substances in Tomatoes under Low-Iron Stress

**Figure 8:**
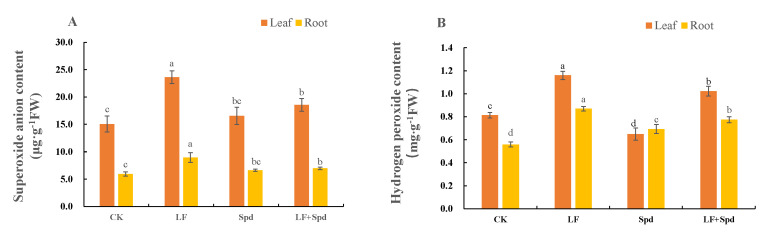
Effect of exogenous Spd on superoxide anion ($O_{2}^{-}$) content and hydrogen peroxide (H_2_O_2_) content under low-iron stress in tomato plants (**A**,**B**). Means denoted by the different lower case letters are significantly different according to Duncan’s multiple range test (*p* ≤ 0.05); the mean represents the average of three replicates and the vertical bar indicates ± standard deviation (SD).

Low-iron stress increased the accumulation of intracellular $O_{2}^{-}$ and H_2_O_2_, leading to increased membrane permeability, and disruption of plant cell membranes as evidenced by a significant increase in the relative electrolyte leakage and MDA content in the root sample. However, exogenous spraying of Spd decreased the $O_{2}^{-}$ and H_2_O_2_ contents, which, in turn, reduced the levels of MDA and relative conductivity, thereby effectively alleviating the deleterious effects of low iron on the cell membrane (Figure 8, Supplementary Figure S3).

**Figure 9:**
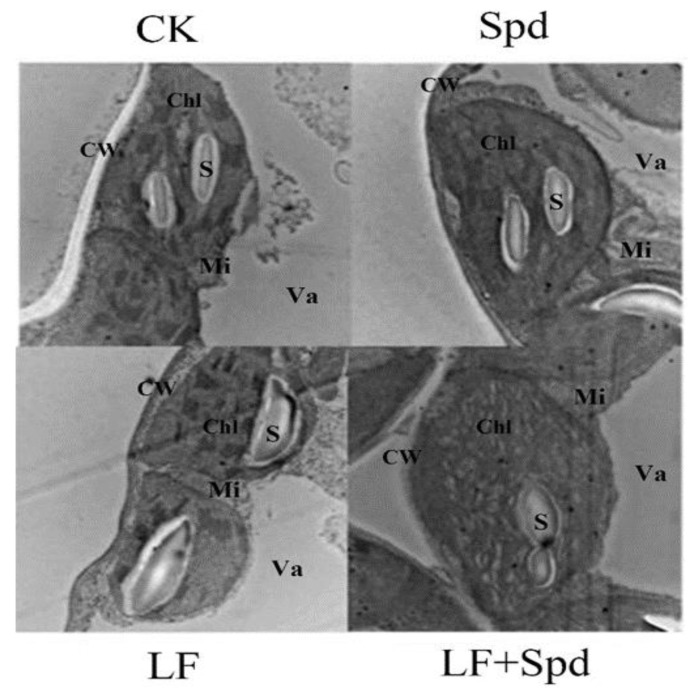
Ultra-structure of leaf cells revealed by transmission electron microscopy. CW, cell wall; Va, vacuole; Chl, chloroplast; Mi, mitochondria; S, starch grain.

Next, we observed the ultrastructure of tomato leaves to reveal the effect of low-iron-induced oxidative stress on the plant cell structure. Figure 9 shows that under low-iron stress, the cell exhibited the phenomenon of plasma-wall separation, the cell membrane was damaged, the chloroplast and starch granules in the leaf were deformed, the chloroplast was irregularly spherical and the starch granule swelled obviously. However, with Spd foliar treatment under low-Fe stress, chloroplast deformity was recovered to some extent with elliptical bands, and the shape of starch grains was restored.

**Figure 10:**
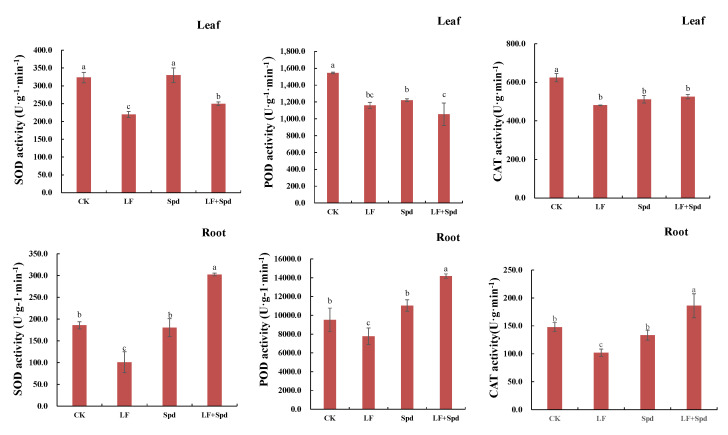
Effect of exogenous Spd on antioxidant enzyme activity under low-iron stress in tomatoes. The first row represents the leaves and the second row represents the roots. Means denoted by the different lower case letters are significantly different according to Duncan’s multiple range test (*p* ≤ 0.05); the mean represents the average of three replicates and the vertical bar indicates ± standard deviation (SD).

To study whether the alleviation of low-iron stress by exogenous Spd was related to the change in antioxidant enzyme activity in tomatoes, we analyzed the activities of SOD, POD and CAT in leaves and roots. The results, shown in Figure 10, revealed that the activities of SOD, POD and CAT decreased in leaves and roots under low-iron stress, which potentially indicated a weakened ROS scavenging ability. However, foliar-spraying with Spd increased the activities of SOD, POD and CAT to varying degrees, thereby effectively alleviating the ROS-induced damage to the cell membrane.

**Figure 11:**
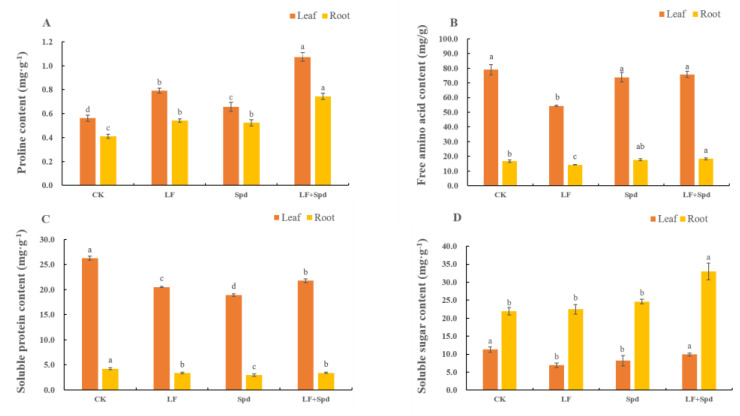
Effect of exogenous Spd on proline content, free amino acid content, soluble protein content and soluble sugar content under low-iron stress in tomato plants (**A**–**D**). Means denoted by the different lower case letters are significantly different according to Duncan’s multiple range test (*p* ≤ 0.05); the mean represents the average of three replicates and the vertical bar indicates ± standard deviation (SD).

We also analyzed the levels of osmoregulatory substances such as proline, sugars and proteins, which are vital for osmotic regulation under stressful conditions in plants. The proline contents in both leaves and roots significantly increased by 40.91 and 32.05%, respectively, and the free amino acid content significantly decreased by 31.20 and 14.79%, respectively, under low-Fe stress when compared with the control. Interestingly, the proline and free amino acid contents in leaves and roots increased with Spd foliar treatment under low-Fe stress compared to low-Fe stress only. The soluble protein content decreased in leaves and roots under low-Fe stress; however, it increased by 13.45% in leaves and 31.16% in roots after Spd foliar treatment under low-Fe stress. The soluble sugar content in leaves significantly decreased by 38.62% under low-Fe stress, while there was no significant change in this in roots. However, compared to low-Fe stress alone, treatment with Spd and low-Fe stress increased the soluble sugar content significantly in both leaves and roots (Figure 11).

### 3.5. Effect of Exogenous Spd on the Organic Acid Content in Roots and the Polyamine Content in Leaves under Low-Iron Stress in Tomato Plants

Oxalic, malic, acetic and citric acids in the root system responded differently to low-Fe stress (Table 6). The oxalic acid level was not significantly altered by low-Fe stress compared to the control; however, the citric and malic acid contents increased by 78.12 and 69.58%, respectively, and the acetic acid content decreased by 49.76% in tomato roots under low-Fe stress. Spd treatment under low-Fe stress significantly increased the contents of citric (49.15%), malic (172.76%) and acetic acids (310.88%) compared to low-Fe stress alone, suggesting that exogenous Spd treatment-induced increased secretion of organic acids from the root potentially enhanced the Fe transport capacity.

Meanwhile, under low-Fe stress, soluble and bound Put, Spd and Spm concentrations increased in the leaves, while free Put decreased and free Spd and Spm did not significantly change (Table 7). It is likely that free polyamines were converted to bound polyamines, which increased the bound polyamines under stress conditions. However, all three forms of polyamines, except for bound Spd, increased to different degrees after Spd foliar-spraying under low-Fe stress. This showed that exogenous Spd treatment could improve the biosynthesis and interconversion of endogenous polyamines to increase the plants’ ability to withstand stress.

### 3.6. Effect of Exogenous Spd on Sugar Metabolism in Tomato Leaves under Low-Iron Stress

**Figure 12:**
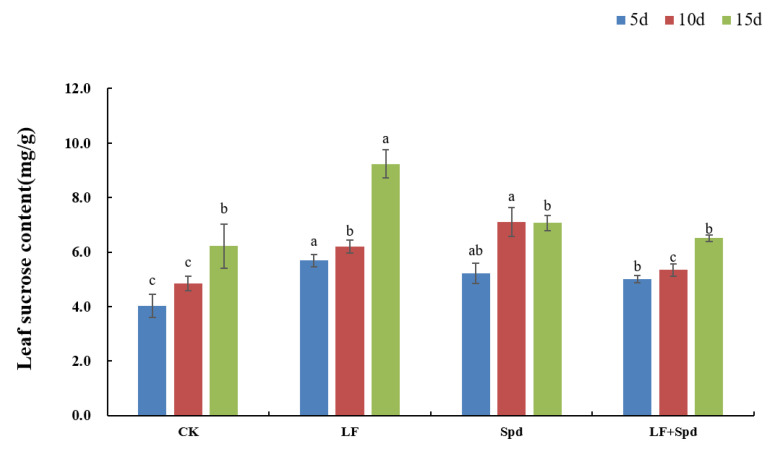
Effects of exogenous Spd on the sucrose content in tomato leaves under low-iron stress. Means denoted by the different lowercase letters on the same color bars are significantly different according to Duncan’s multiple range test (*p* ≤ 0.05); the mean represents the average of three replicates and the vertical bar indicates ± standard deviation (SD).

In addition to being a source of energy for plant metabolism, sucrose has also been identified as a signaling molecule involved in the regulation of Fe deficiency. The sucrose content in leaves increased by 41.52, 28.24 and 48.57% with time after low-Fe treatment, and was higher than the control. Spd-spraying under low-Fe stress significantly reduced the sucrose content in the leaves compared to the low-Fe treatment (Figure 12).

The results of the measurement of enzymes’ activities related to sugar metabolism showed that under low-iron stress, the activities of both SS and SPS enzymes decreased, while the activities of two conversion enzymes, NI and AI, increased on day 10 after low-Fe treatment, although the sucrose content increased rather than decreased. This shows that the catabolic direction of SS and SPS enzyme activities was greater than the synthetic direction under low-Fe stress, and with the decrease in enzyme activities, the transport of photosynthetic products was blocked, causing the accumulation of sucrose in leaves, while the degradation and utilization of sucrose were weakened, which, in turn, stimulated the activities of two converting enzymes, NI and AI, and maintained the stability of sucrose anabolism. Exogenous Spd treatment significantly increased the SS activity and decreased the sucrose content, indicating that Spd promotes the degradation of sucrose, accelerates the consumption of sucrose transported from the leaves, promotes the transfer of photosynthetic products from the source to the reservoir and prevents the inhibitory effect of sucrose accumulation on photosynthetic efficiency (Supplementary Table S4).

## 4. Discussion

Iron is a vital element for the metabolism, growth and development of plants. Nevertheless, the lack of adaptive mechanisms to combat iron deficiency severely impairs plant biomass accumulation. Biomass is a direct manifestation of plant growth variation and can be an important basis for assessing the degree of plant injury due to stress [45]. Roots not only provide structural support to the above-ground parts of the plant but also provide nutrients and water. Therefore, the survival of a plant depends on its proper growth, development and root function [46]. Under low-iron stress, a decrease in above-ground and below-ground biomass (Table 2), and a suppressed total root length, total root surface area and total root volume of seedlings were observed (Supplementary Table S3). Morphological inhibition is one of the adverse effects caused by low-iron stress, and our results were consistent with earlier accounts of iron-deficiency effects on crop plants [47]. This is because adverse stress conditions inhibit both the division and growth of root cells, causing a significant decline in root biomass [48]. However, foliar-spraying with Spd increased not only above-ground and below-ground biomass but also the total root length, total root surface area, total root volume, root vigor and Fe^3+^ reductase activity, which potentially improved nutrient acquisition and alleviated low-iron stress in tomato seedlings.

Since photosynthesis is the most essential plant process, its efficiency has a significant influence on growth, yield and stress resistance in plants [49]. In this study, the photosynthetic pigment content of tomato leaves was significantly inhibited under low-iron stress and leaf photosynthetic activity was drastically reduced (Table 3 and Table 4), which is consistent with the findings of Yao et al. [50]. This is because iron-deficiency stress hinders chlorophyll synthesis in tomato seedlings, leading to a reduction in chloroplast lamellae and disruption of the chloroplast structure. However, the chlorophyll contents in tomato leaves increased significantly after Spd foliar-spraying. Such effects support the hypothesis that the ability to capture and convert light energy was restored, and the exogenously sprayed Spd could safeguard chloroplasts and protect the photosynthetic mechanism from the adverse effects of environmental stress [51]. Moreover, chlorophyll fluorescence parameters such as Fv/Fm, PSII, ETR, etc., decreased significantly and NPQ increased under low-iron stress (Table 5), which was in agreement with the previous findings [52]. This is because damage to the photosystem II reaction centered on low-iron stress-inhibited PS II photochemical activity, reduced PS II primary light energy conversion efficiency and hindered the photosynthetic electron transfer process. Consistent with the previous reports in Sweet Corn [53], exogenous Spd increased the chlorophyll content and stabilized the photosynthetic system in tomato seedlings, thus alleviating the damage to the photosystem and enhancing or restoring photosynthetic efficiency. It can be inferred that exogenous Spd-spraying is crucial to improve the photosynthetic efficiency of tomato seedlings, leading to increased biomass and dry matter accumulation.

Polyamines protect plants from environmental stress by regulating the accumulation of sugar, proline and other osmotic substances [54]. Proline is an important osmotic adjustment substance in plants that functions in maintaining the membrane structure and is used as a physiological and biochemical indicator for the plant stress response [55]. Du [56] showed that the proline content in plants under stress increased, and was further increased by Spd treatment, which is in agreement with our results showing that proline content in leaves and roots of tomato seedlings under low-iron stress increased significantly compared to the control, and were further significantly increased after foliar-spraying of Spd under low-iron stress compared to LF treatment. The proteins synthesized and stored during plant growth are degraded to free amino acids for biosynthesis to maintain normal plant life activities [57]. When plants are subjected to stress, particularly osmotic stress, the soluble sugar content increases, which can improve the osmoregulatory capacity of leaves and provide carbon and nitrogen sources for plant organic matter synthesis [58]. The soluble sugar content in leaves and roots of tomato seedlings decreased under low-iron stress; however, exogenous Spd treatment increased the soluble sugar content in tomato seedlings under low-iron stress, suggesting that Spd improves the ability of plants to synthesize sugars [59]. To improve the plant tolerance to iron deficiency, roots can reduce the inter-root pH by secreting organic acids and increasing Fe^3+^ solubility [60]. Exogenous spraying with Spd significantly increased the content of citric and malic acids in the root system, which indicates that Spd potentially increases the secretion of organic acids in the root system, thus enhancing the iron transport in plants [61].

Plant performance under multiple abiotic stresses is linked to the accumulation of Put, Spd and Spm [62]. In this study, the content of all three forms of polyamines increased to different degrees after Spd-spraying, which is consistent with the results of Shan et al. [61]. It is highly likely that exogenous Spd treatment potentially improves the biosynthesis of endogenous polyamines and significantly enhances the ability of plants to withstand adversity. Moreover, the study also found that Spd treatment significantly increased SS enzyme activity, reduced sucrose content, promoted sucrose degradation, accelerated sucrose consumption, facilitated the transfer of photosynthetic products from source to sink and prevented the inhibitory effect of sucrose accumulation on photosynthetic efficiency in tomato seedlings [63], which is consistent with the results of our study.

Under stress conditions, reactive oxygen species (ROS) are profusely generated in plants, causing oxidative stress and damage to important molecules in plants [55,64]. The cell membrane is a barrier that maintains the relative stability of plant cells. Under stress conditions, the degree of membrane lipid peroxidation intensifies due to excessive accumulation of ROS, which changes the membrane permeability and affects the normal physiological and biochemical reactions [65]. In this study, low-iron stress reduced SOD, POD and CAT activities in tomato plants and weakened their ability to scavenge ROS, resulting in excessive intracellular $O_{2}^{-}$ and H_2_O_2_ accumulation, increased membrane permeability and disruption of plant cell membranes (Figure 8). This relies on the fact that iron acts as a component of enzymes such as SOD, POD and CAT, and the three enzymes’ activities were significantly inhibited when plants were subjected to a low-iron environment. After exogenous spraying of Spd treatment, the SOD, POD and CAT activities increased to different degrees and $O_{2}^{-}$, H_2_O_2_, MDA and the relative conductivity decreased, indicating that Spd effectively alleviated the extent of cell membrane disruption.

In iron-chelating reductase *FRO7* mutant plants, the iron content in chloroplasts and the activity of iron reductase are significantly lower than in wild-type plants, and the electron transport chain in the photosystem is interrupted, causing impaired photosynthesis [66]. Moreover, *FRO7* mutant plants show a severe yellowing phenotype, along with the occurrence of seedling lethality, indicating that the *FRO7* gene is important for maintaining iron homeostasis in chloroplasts and for the proper performance of photosynthesis in the plant [66]. In the present study, Spd treatment under low-iron stress upregulated the expression of the *FRO* gene and related Fe transporter genes *IRT1* and *IRT2* in the root, which is consistent with the results of a previous study in *Pyrus betulaefolia* [67].

Previous studies established that IAA plays an important role as a signaling molecule in the response to iron deficiency in plants, and that the local iron supply affects the plant lateral root growth and development by inducing the growth hormone AUX-1 transporter [68]. The strategy-I plants induce ethylene synthesis in response to iron-deficiency stress, and ethylene positively regulates the iron-deficiency response [69]. The ethylene response factor *ERF4/ERF72* is involved in iron-deficiency response in apple rootstocks, and interference with these two genes results in upregulated expression of iron-uptake genes in *Ziziphus jujube* roots, promoting iron uptake by the roots [70]. Accordingly, we also found that transcript levels of *ERF1* and *ERF2* genes were upregulated in the root system under low-iron stress, and exogenous spraying of Spd treatment further upregulated the expression of *ERF1* genes in the leaves, while it downregulated them in the root. Differential expression of these genes related to growth hormones and ethylene, together with the expression of downstream *FRO* and *IRT1* genes, potentially contributed to improved iron acquisition and transport under low-iron stress.

Meanwhile, sucrose accumulation in leaves increased under low-iron stress, which indicated that the translocation capacity of sucrose to the root system was possibly reduced; nonetheless, sucrose could act as a long-range signal to regulate the response of plants to Fe deficiency [71]. It is worth noting at this point that the expression of genes such as *COX15* in chlorophyll metabolism was downregulated under low-iron stress, indicating that the transport of sucrose to the lower part of the ground was inhibited [72]. The upregulated expression of genes such as *CHLP*, *PetF* and *CAT*, which are involved in chlorophyll synthesis and antioxidant enzyme activities, as well as significantly upregulated *SUS* and *TPS* gene expression and significantly increased sucrose synthase activity after Spd-spraying, indicated that Spd treatment also affected sugar metabolism to confer tolerance to low-iron stress in tomato plants.

## 5. Conclusions

Iron (Fe) deficiency severely limits agricultural crop yield due to its low availability, particularly in soils with a high pH. The success of iron fertilization largely depends on soil pH management, which is very challenging in field conditions. In this study, we showed that foliar application of exogenous plant growth regulator Spd could improve plant tolerance to low-iron stress. Briefly, the transcriptomic analysis revealed that exogenous Spd could regulate the plant response to low-iron stress by modulating the expression of genes involved in the processes of hormone metabolism, sucrose metabolism, antioxidant defense system, photosynthesis, chlorophyll metabolism and Fe uptake and transport. Besides this, biochemical and physiological analyses revealed that low-iron stress-induced suppression, in photosynthesis and growth of tomato seedlings, were significantly alleviated by exogenous Spd treatment, which was closely associated with differential modulation of photosynthetic pigment contents, gas exchange, chlorophyll fluorescence capacity, proline content, sucrose content, root vigor, citric and malic acid contents, ROS metabolism and polyamine synthesis and interconversion. Overall, this study reveals the critical mechanism of exogenous Spd-induced enhanced tolerance to low-iron stress in tomatoes and provides a novel characterization of the key traits associated with the adaptation of tomatoes to a low-iron environment. Traits associated with changes in low-iron-tolerance genes can potentially be used to improve yields of greenhouse tomatoes in low-iron environments. Nonetheless, large-scale experimentation is required to unveil and extend this knowledge, to develop better agricultural practices.

## Figures and Tables

**Table 1:** **Statistics of transcriptome sequencing data.** Sample, sample name; Raw Read, the number of reads in total; Valid Read, the number of valid reads after de-junctioning, de-low quality, etc.; Valid Ratio, the proportion of valid reads; Mapped Reads, the number of reads that can be compared to the genome; Unique Mapped Reads, can only uniquely match to one position in the genome; Q20%, the percentage of bases with Q20% quality value ≥ 20 (sequencing error rate less than 0.01); Q30%, the percentage of bases with Q30% quality value ≥ 30 (sequencing error rate less than 0.001).

Sample	Raw Read	Valid Read	Valid Ratio (Reads)	Mapped Reads	Unique Mapped Reads	Q20%	Q30%
**CK_L1**	51,425,238	47,618,720	92.60	45,445,100 (95.44%)	38,907,284 (81.71%)	99.99	97.68
**CK_L2**	41,647,086	39,867,334	95.73	38,103,955 (95.58%)	32,333,283 (81.10%)	99.99	97.78
**CK_L3**	36,237,924	34,971,030	96.50	33,505,416 (95.81%)	28,442,929 (81.33%)	99.99	97.59
**CK_R1**	41,884,204	40,904,806	97.66	35,303,817 (86.31%)	30,123,337 (73.64%)	99.99	97.38
**CK_R2**	47,810,190	46,772,676	97.83	42,676,467 (91.24%)	36,960,152 (79.02%)	99.98	98.37
**CK_R3**	51,562,904	50,495,438	97.93	44,954,365 (89.03%)	38,867,119 (76.97%)	99.98	98.30
**LF_L1**	45,353,480	42,425,970	93.55	40,385,907 (95.19%)	34,478,301 (81.27%)	99.99	97.79
**LF_L2**	45,112,774	43,318,042	96.02	41,436,986 (95.66%)	35,189,847 (81.24%)	99.99	97.62
**LF_L3**	47,262,530	45,751,138	96.80	43,773,386 (95.68%)	37,222,647 (81.36%)	99.99	97.73
**LF_R1**	43,262,284	42,427,286	98.07	39,214,339 (92.43%)	33,844,982 (79.77%)	99.99	98.39
**LF_R2**	52,854,702	51,760,038	97.93	47,780,225 (92.31%)	41,266,840 (79.73%)	99.99	98.51
**LF_R3**	52,525,326	51,551,094	98.15	45,715,483 (88.68%)	39,276,860 (76.19%)	99.99	98.45
**LFS_L1**	46,825,170	44,333,134	94.68	42,409,857 (95.66%)	36,246,131 (81.76%)	99.99	97.50
**LFS_L2**	35,688,744	34,009,554	95.29	32,356,247 (95.14%)	27,660,644 (81.33%)	99.99	97.25
**LFS_L3**	41,306,858	39,800,370	96.35	38,018,606 (95.52%)	32,451,594 (81.54%)	99.99	97.60
**LFS_R1**	53,734,354	52,663,752	98.01	47,503,814 (90.20%)	40,935,810 (77.73%)	99.99	98.41
**LFS_R2**	52,372,096	51,298,494	97.95	45,352,741 (88.41%)	39,262,642 (76.54%)	99.98	98.37
**LFS_R3**	54,358,180	53,210,976	97.89	48,848,829 (91.80%)	42,040,996 (79.01%)	99.99	98.43
**Spd_L1**	50,060,896	45,020,762	89.93	43,045,480 (95.61%)	36,727,980 (81.58%)	99.99	97.52
**Spd_L2**	35,827,860	34,695,530	96.84	33,367,320 (96.17%)	28,459,478 (82.03%)	99.99	97.74
**Spd_L3**	50,682,352	48,290,700	95.28	46,229,329 (95.73%)	39,485,340 (81.77%)	99.99	97.53
**Spd_R1**	52,910,480	51,911,130	98.11	46,563,164 (89.70%)	40,136,000 (77.32%)	99.98	98.42
**Spd_R2**	51,487,988	50,453,420	97.99	45,578,298 (90.34%)	39,515,003 (78.32%)	99.98	98.44
**Spd_R3**	53,683,760	52,593,000	97.97	46,823,515 (89.03%)	40,562,480 (77.13%)	99.98	98.48

**Table 2:** Effects of exogenous Spd on tomato biomass under low-iron stress.

Treatments	Shoot Fresh Weight/g	Root Fresh Weight/g	Shoot Dry Weight/g	Root Dry Weight/g	Total Fresh Weight/g	Total Dry Weight/g
**CK**	5.43 ± 0.44 ab	1.45 ± 0.12 b	0.40 ± 0.01 b	0.09 ± 0.01 a	6.88 ± 0.43 b	0.49 ± 0.02 b
**LF**	3.80 ± 0.61 c	1.16 ± 0.13 c	0.29 ± 0.05 c	0.06 ± 0.01 b	4.96 ± 0.73 c	0.35 ± 0.03 c
**Spd**	6.19 ± 0.45 a	1.81 ± 0.12 a	0.48 ± 0.03 a	0.10 ± 0.01 a	7.99 ± 0.43 a	0.58 ± 0.03 a
**LF + Spd**	5.07 ± 0.24 b	1.42 ± 0.19 bc	0.36 ± 0.02 b	0.08 ± 0.01 a	6.49 ± 0.35 b	0.45 ± 0.01 b

CK, control; LF, Low Fe; Spd, spermidine; LF + Spd, Low Fe plus spermidine. Data are shown as mean ± SD. Within each column, entries followed by the same lowercase letters are not significantly different according to Duncan’s test at *p* ≤ 0.05.

**Table 3:** Effects of exogenous Spd on chlorophyll content in tomato leaves under low-iron stress.

Treatments	Chl a mg·g^−1^ FW	Chl b mg·g^−1^ FW	Carotenoid mg·g^−1^ FW	Chl a + b mg·g^−1^ FW
**CK**	1.47 ± 0.06 b	0.66 ± 0.02 b	0.24 ± 0.02 a	3.20 ± 0.08 b
**LF**	1.06 ± 0.03 d	0.56 ± 0.04 c	0.15 ± 0.01 b	2.41 ± 0.08 d
**Spd**	1.64 ± 0.02 a	0.70 ± 0.01 a	0.25 ± 0.02 a	3.52 ± 0.07 a
**LF + Spd**	1.31 ± 0.10 c	0.63 ± 0.02 b	0.18 ± 0.02 b	2.93 ± 0.16 c

CK, control; LF, Low Fe; Spd, spermidine; LF + Spd, Low Fe plus spermidine. Data are shown as mean ± SD. Within each column, entries followed by the same lowercase letters are not significantly different according to Duncan’s test at *p* ≤ 0.05.

**Table 4:** Effects of exogenous Spd on photosynthetic parameters in tomato leaves under low-iron stress.

Treatments	Pn/ (μmol·m^−1^·s^−1^)	Gs/ (mmol·m^−1^·s^−1^)	Ci/ (mmol·mol^−1^)	Tr/ (mmol·m^−1^·s^−1^)
**CK**	7.56 ± 0.10 b	180.55 ± 1.68 a	130.91 ± 1.58 c	3.55 ± 0.00 a
**LF**	3.85 ± 0.08 d	92.73 ± 1.55 c	136.26 ± 2.55 b	2.04 ± 0.02 c
**Spd**	8.92 ± 0.03 a	178.94 ± 1.28 a	122.71 ± 0.80 d	3.54 ± 0.02 a
**LF + Spd**	4.73 ± 0.12 c	139.87 ± 0.43 b	153.42 ± 1.47 a	2.90 ± 0.05 b

CK, control; LF, Low Fe; Spd, spermidine; LF + Spd, Low Fe plus spermidine. Data are shown as mean ± SD. Within each column, entries followed by the same lowercase letters are not significantly different according to Duncan’s test at *p* ≤ 0.05.

**Table 5:** Effects of exogenous Spd on fluorescence parameters in tomato leaves under low-iron stress.

Treatments	Fv/Fm	ETR	ΦPSII	qP	NPQ
**CK**	0.763 ± 0.007 b	120.562 ± 0.95 b	0.276 ± 0.002 b	0.468 ± 0.026 b	1.380 ± 0.038 c
**LF**	0.706 ± 0.009 d	75.706 ± 3.14 d	0.173 ± 0.007 d	0.302 ± 0.012 d	2.566 ± 0.056 a
**Spd**	0.776 ± 0.008 a	146.958 ± 0.77 a	0.337 ± 0.002 a	0.518 ± 0.004 a	1.314 ± 0.012 c
**LF + Spd**	0.741 ± 0.002 c	106.407 ± 1.21 c	0.243 ± 0.003 c	0.416 ± 0.006 c	1.851 ± 0.022 b

CK, control; LF, Low Fe; Spd, spermidine; LF + Spd, Low Fe plus spermidine. Data are shown as mean ± SD. Within each column, entries followed by the same lowercase letters are not significantly different according to Duncan’s test at *p* ≤ 0.05.

**Table 6:** Effects of exogenous Spd on organic acid content in tomato roots under low-iron stress.

Treatments	Oxalic Acid/(mg·g^−1^)	Malic Acid/(µg·g^−1^)	Citric Acid/(µg·g^−1^)	Acetic Acid/(µg·g^−1^)
**CK**	2.68 ± 0.13 a	357.77 ± 28.45 c	90.95 ± 4.85 c	114.52 ± 15.87 c
**LF**	2.73 ± 0.13 a	606.70 ± 97.39 bc	162.00 ± 23.73 b	57.54 ± 12.40 d
**Spd**	2.24 ± 0.09 b	684.47 ± 167.26 b	133.55 ± 9.60 bc	210.01 ± 11.88 b
**LF + Spd**	2.75 ± 0.35 a	1654.85 ± 218.26 a	241.63 ± 40.10 a	236.42 ± 15.10 a

CK, control; LF, Low Fe; Spd, spermidine; LF + Spd, Low Fe plus spermidine. Data are shown as mean ± SD. Entries within each column followed by the same lowercase letters are not significantly different according to Duncan’s test at *p* ≤ 0.05.

**Table 7:** Effects of exogenous Spd on the polyamine content in tomato leaves under low-iron stress.

Treatments	Free Polyamine (nmol·g^−1^)			Soluble Conjugated Polyamine (nmol·g^−1^)			Bound Polyamine (nmol·g^−1^)		
	Put	Spd	Spm	Put	Spd	Spm	Put	Spd	Spm
**CK**	662.64 ± 16.76 b	429.20 ± 57.33 b	142.69 ± 1.68 b	111.21 ± 39.69 c	30.44 ± 3.91 d	60.37 ± 2.54 c	828.56 ± 91.03 c	238.62 ± 27.29 c	451.48 ± 36.43 c
**LF**	545.76 ± 43.10 c	568.52 ± 43.73 b	160.10 ± 3.17 ab	229.51 ± 46.45 b	85.60 ± 4.36 b	96.29 ± 2.73 b	6909.74 ± 433.32 a	2064.22 ± 130.05 a	794.57 ± 43.54 b
**Spd**	848.99 ± 64.31 a	574.61 ± 30.55 b	160.37 ± 13.66 ab	129.79 ± 18.81 c	55.02 ± 4.26 c	63.07 ± 2.60 c	1060.74 ± 47.86 c	308.22 ± 14.31 c	496.38 ± 18.08 c
**LF + Spd**	702.82 ± 55.56 b	878.03 ± 140.96 a	170.73 ± 12.87 a	408.15 ± 78.32 a	103.22 ± 4.38 a	119.36 ± 9.19 a	4963.23 ± 340.45 b	1479.83 ± 102.24 b	1103.26 ± 55.84 a

CK, control; LF, Low Fe; Spd, spermidine; LF + Spd, Low Fe plus spermidine. Data are shown as mean ± SD. Entries within each column followed by the same lowercase letters are not significantly different according to Duncan’s test at *p* ≤ 0.05.

## Data Availability

Data are contained within the article and Supplementary Materials.

## Supplementary Materials

The following supporting information can be downloaded at: https://www.mdpi.com/article/10.3390/antiox11071260/s1, Figure S1: The relative expression of selected differentially expressed genes verified by qRT-PCR; Figure S2: Effect of exogenous Spd on (A) root vigor and (B) Fe^3+^ reductase activity of tomato under low-iron stress; Figure S3: Effect of exogenous Spd on lipid peroxidation (A) and ion leakage (B) in tomato roots under low-iron stress; Table S1: qRT-PCR test reaction system; Table S2: Primers used for qRT-PCR; Table S3: Effects of exogenous Spd on tomato root morphological indexes under low-iron stress; Table S4: Effects of Spd on enzyme activities related to sucrose metabolism in tomato leaves under low-iron stress.
