# Supplementary material for: Comparative Physiological and Transcriptomic Analyses Reveal Mechanisms of Exogenous Spermidine-Induced Tolerance to Low-Iron Stress in Solanum lycopersicum L
Source: Antioxidants (Basel). 2022 Jun 27;11(7):1260. doi: 10.3390/antiox11071260 (PMC9312307; doi:10.3390/antiox11071260)
Supplement: Supplementary file 1 [file antioxidants-11-01260-s001.zip › Supplementary figures.pdf]

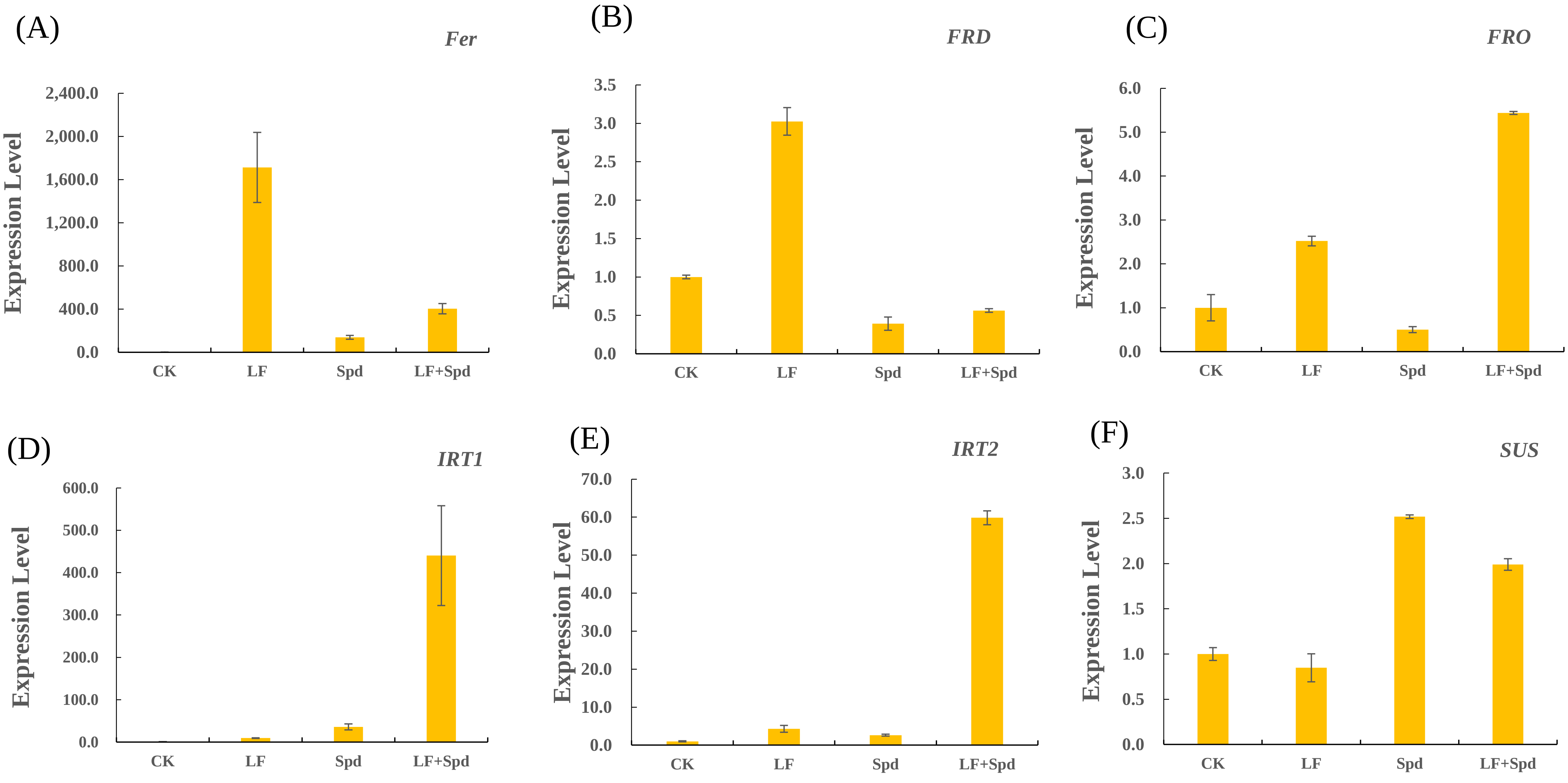

Supplementary figure S1. The relative expression of selected differentially expressed genes verified by qRT-PCR.

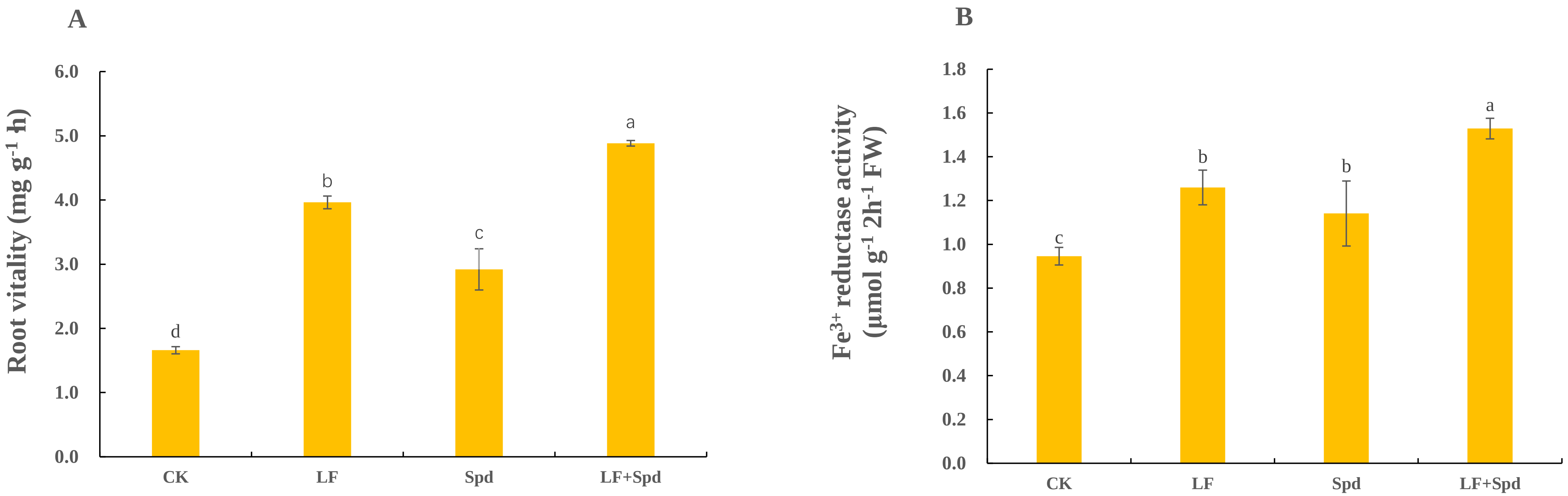

Supplementary figure S2. Effect of exogenous Spd on (A) root vigor and (B) Fe^{3+} reductase activity of tomato under low iron stress.

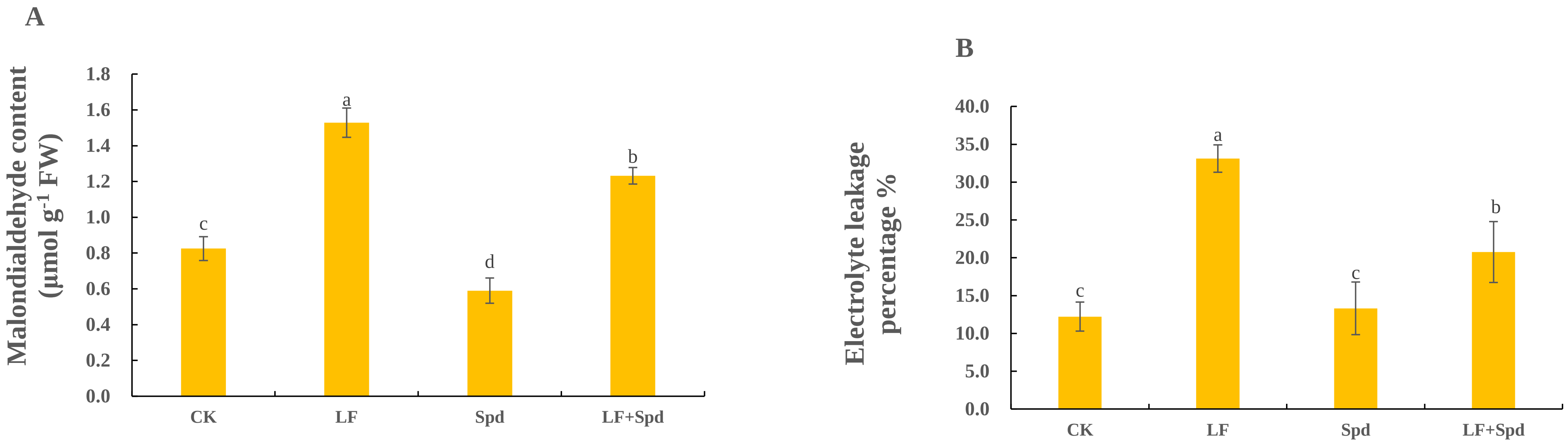

Supplementary figure S3. Effect of exogenous Spd on lipid peroxidation (A) and ion leakage (B) in tomato roots under low iron stress.
