# Supplementary material for: Comparative Physiological and Transcriptomic Analyses Reveal Mechanisms of Exogenous Spermidine-Induced Tolerance to Low-Iron Stress in Solanum lycopersicum L
Source: Antioxidants (Basel). 2022 Jun 27;11(7):1260. doi: 10.3390/antiox11071260 (PMC9312307; doi:10.3390/antiox11071260)
Supplement: Supplementary file 1 [file antioxidants-11-01260-s001.zip › Supplementary tables.pdf]

Supplementary Table S1 qRT-PCR test reaction system

Reactant	concentration	volume（μL）
**ChamQ Universal SYBR Color qPCR Master Mix**	2X	10
**Primers-F**	10μM	0.4
**Primers-R**	10μM	0.4
**Template（cDNA）**		2

Supplementary Table S2 Primers used for qRT-PCR

Gene	Accession No.	Forward primer	Reverse primer
FRO	*Solyc04g071610*	GGAGCCAGAGAAAATCAGTG	CGAAGCCATAGGAGTTGC
IRT2	*Solyc02g069190*	AATCCAGAAACTGGTGGTGCTG	GAAAAGTATACACGATTACAATTTTGC
FRD3	*Solyc11g013440*	TCCATTGGGGTGTCAGTGGTG	CTGCGTTGTGCCTAATCGTGC
IRT1	*Solyc02g069200*	TGGCTGTGGCTGGAAATCATGTTC	AGAATTTTTTTGCAACTCCCAATAGGT
Fer	*Solyc12g008740*	TGTTTTTCCGCAGCCAATG	TGCCAAAGTTTATGTCCACCTCA
SUS	*Solyc12g009300*	GAAAGAGGATGGGGAAACACT	AAGGATGCGAGGGACGATA
Actin	*Solyc03g078400*	ACCACTGAGCACAATGTTACCG	GTCCTCTTCCAGCCATCCA

Supplementary Table S3 Effects of exogenous Spd on tomato root morphological indexes under low iron stress

Treatment	Total root length /cm	Total root surface area /cm^2	Average diameter /mm	Total root volume /cm^3
**CK**	313.34±15.33a	61.52±9.55a	0.68±0.02b	1.21±0.14a
**LF**	129.20±17.52c	34.49±4.18d	0.85±0.05a	0.66±0.04d
**Spd**	322.80±43.19a	58.94±7.31b	0.64±0.08c	1.11±0.15ab
**LF+Spd**	230.79±49.10b	48.75±8.67c	0.68±0.09b	0.93±0.04c

Supplementary Table S4. Effects of Spd on enzyme activities related to sucrose metabolism in tomato leaves under low iron stress

Treatments	AI (U g^{-1}FW)			NI (U g^{-1}FW)			SS (U g^{-1}FW)			SPS (U g^{-1}FW)		
	5 d	10 d	15 d	5 d	10 d	15 d	5 d	10 d	15 d	5 d	10 d	15 d
CK	259.59 ±17.44a	158.79 ±11.61b	170.69 ±2.67c	63.01 ±4.85b	43.18 ±1.74c	23.10 ±1.12b	2584.24 ±220.20b	2943.75 ±177.86b	1907.59 ±150.15a	170.60 ±39.17b	384.55 ±13.20a	558.77 ±21.51a
LF	203.64 ±17.28b	236.39 ±27.81a	219.75 ±22.08a	98.63 ±6.77a	48.96 ±0.98b	16.85 ±1.08c	2046.53 ±131.33c	1901.43 ±206.80d	2081.98 ±255.59b	106.19 ±14.44c	348.74 ±19.70b	508.58 ±13.97bc
Spd	198.05 ±8.88bc	148.75 ±16.27b	192.46 ±2.88b	50.74 ±2.31c	42.34 ±0.39c	28.16 ±1.76a	3738.05 ±84.11a	3447.18 ±103.87a	3638.46 ±86.19c	111.85 ±13.45c	372.78 ±7.68ab	543.14 ±27.63ab
LF+Spd	172.14 ±14.64c	163.53 ±10.63b	93.60 ±5.42d	51.90 ±0.45c	55.70 ±1.47a	23.20 ±1.20b	2542.83 ±52.77b	2542.02 ±147.01c	2789.34 ±134.18c	381.92 ±37.72a	378.15 ±18.62ab	502.27 ±6.09c
